# Genome-wide association studies for production, respiratory disease, and immune-related traits in Landrace pigs

**DOI:** 10.1038/s41598-021-95339-2

**Published:** 2021-08-04

**Authors:** Yoshinobu Uemoto, Kasumi Ichinoseki, Toshimi Matsumoto, Nozomi Oka, Hironori Takamori, Hiroshi Kadowaki, Chihiro Kojima-Shibata, Eisaku Suzuki, Toshihiro Okamura, Hisashi Aso, Haruki Kitazawa, Masahiro Satoh, Hirohide Uenishi, Keiichi Suzuki

**Affiliations:** 1 Graduate School of Agricultural Science, Tohoku University, Sendai, Miyagi 980-8572 Japan; 2 Animal Bioregulation Unit, Division of Animal Sciences, Institute of Agrobiological Sciences, National Agriculture and Food Research Organization (NARO), Tsukuba, Ibaraki 305-8634 Japan; 3 Miyagi Prefecture Animal Industry Experiment Station, Osaki, Miyagi 989-6445 Japan; 4 Institute of Livestock and Grassland Science, NARO, Tsukuba, Ibaraki 305-0901 Japan

**Keywords:** Animal breeding, Genome-wide association studies, Quantitative trait

## Abstract

Identification of a quantitative trait locus (QTL) related to a chronic respiratory disease such as Mycoplasmal pneumonia of swine (MPS) and immune-related traits is important for the genetic improvement of disease resistance in pigs. The objective of this study was to detect a novel QTL for a total of 22 production, respiratory disease, and immune-related traits in Landrace pigs. A total of 874 Landrace purebred pigs, which were selected based on MPS resistance, were genotyped using the Illumina PorcineSNP60 BeadChip. We performed single nucleotide polymorphism (SNP)-based and haplotype-based genome-wide association studies (GWAS) to detect a novel QTL and to evaluate the possibility of a pleiotropic QTL for these traits. SNP-based GWAS detected a total of six significant regions in backfat thickness, ratio of granular leucocytes to lymphatic cells, plasma concentration of cortisol at different ages, and complement alternative pathway activity in serum. The significant region detected by haplotype-based GWAS was overlapped across the region detected by SNP-based GWAS. Most of these detected QTL regions were novel regions with some candidate genes located in them. With regard to a pleiotropic QTL among traits, only three of these detected QTL regions overlapped among traits, and many detected regions independently affected the traits.

## Introduction

*Mycoplasma hyopneumoniae* (Mhp) is considered to play a primary role in porcine respiratory disease complex and Mycoplasmal pneumonia of swine (MPS) is a chronic respiratory disease caused by Mhp^1^. Chronic respiratory diseases caused by Mhp are associated with serious economic losses in the pig industry as they may result in decreased growth performance and feed efficiency and, sometimes, increased mortality rates^2^. Therefore, it is important to prevent Mhp infection and maintain animal health in the pig industry. Strategies for the control of MPS include improving housing conditions and providing antimicrobial medication and vaccination^2^. However, the complicated mechanisms of Mhp infection and its co-infection with other respiratory pathogens make it difficult to maintain high performance by controlling the spread of MPS in the pig industry.

Recently, the genetic improvement of disease resistance—genetic selection for resistance to disease by selection of a disease-resistance indicator such as infection rate or immune response—has been well-received. For example, Kadowaki et al.^3^ reported a selection-based closed-line breeding experiment carried out in Landrace purebred pigs over five generations to improve MPS resistance using the aggregate breeding value of their production traits and the MPS lesion score (MPS score). The MPS-selected Landrace line showed significantly lower MPS score than that of a non-selected Landrace line^4^, which confirmed the effectiveness of genetic selection based on infection rate such as the MPS score. In addition, Okamura et al.^5^ and Sato et al.^6^ also evaluated the correlated responses of immune capacity traits and peripheral blood cytokines in MPS-selected pigs, and observed the indirect selection of these immune-related traits due to the genetic decrease in the MPS score. These authors suggested that, not only the infection rate, but also immune-related traits can be useful indicators for genetic improvement of disease resistance in pigs.

Identification of a quantitative trait locus (QTL) related to respiratory disease and immune-related traits is important for understanding the genomic background of disease resistance and to apply marker-assisted selection as a selection indicator. A close-line breeding population is useful as a target population for detecting a significant QTL because changes in allele frequencies associated with selection traits can occur in this population^7–9^. Okamura et al.^10^ reported several significant QTLs for respiratory disease and immune-capacity traits via linkage-based QTL mapping using microsatellite markers in MPS-selected pigs. A high-density single nucleotide polymorphism (SNP) array has recently made it possible to effectively detect a significant QTL using genome-wide association studies (GWAS). Sato et al.^9^ reported that, in a simulation analysis in a close-line breeding population, the power of SNP-based GWAS was greater than that of haplotype-based GWAS—a linkage-based method wherein haplotypes are constructed based on pedigree and linkage disequilibrium (LD) information. Therefore, a novel significant QTL for respiratory disease and immune-related traits could be detected by SNP-based GWAS in such populations.

The objective of this study was (1) to detect a novel QTL for production, respiratory disease and immune-related traits in MPS-selected pigs by performing SNP-based GWAS, and (2) to evaluate the possibility of a pleiotropic QTL for these traits by multi-trait meta-analysis. We also performed haplotype-based GWAS to compare with the results of SNP-based GWAS.

## Materials and methods

### Experimental animals and phenotyping

All animals were cared for and slaughtered according to Japanese animal welfare regulations. Animal Care and Use Committee approval was not obtained for this study because the phenotype and pedigree data were obtained from an existing database^3,5,6^ and DNA samples for DNA^10^ were used for SNP genotyping.

A complete description of the experimental population was previously reported by Kadowaki et al.^3^, Okamura et al.^5,10^, and Sato et al.^6^. In brief, Landrace purebred pigs, selected over five generations from 2002 to 2008 at the Miyagi Prefecture Livestock Experimental Station, Japan, were used in this study. This population was selected based on an average daily gain (DG105) from 30 to 105 kg of body weight (BW), ultrasound backfat thickness at 105 kg BW (BF), MPS score, and plasma concentrations of cortisol at 105 kg BW (CORT_105). The pigs were infected with respiratory diseases under natural conditions. The detailed selection method and procedure for measuring traits has been described by Kadowaki et al.^3^.

The phenotypes were measured for pigs from the first generation (G1) to the fifth generation (G5). Production and immune-related traits were measured for the selection candidates, and production, respiratory disease, and immune-related traits were measured for their slaughtered full-sib pigs as sib-tested pigs. The sib-tested pigs were slaughtered at 105 kg of BW, and their lungs and snouts were then immediately assessed to determine the MPS score and the atrophic rhinitis score (AR score), respectively. A total of 931 pigs, six generations from the base generation (G0) to G5, were used as the pedigree information, and the population comprised one family (in a complex multigenerational pedigree) such that all individuals were related. Table 1 shows the number of pigs including the selection candidates and sib-tested pigs per generation. A total of 874 Landrace purebred pigs, which were 622 sib-tested pigs, 214 parent pigs, and 38 unselected pigs, were used for SNP genotyping in this study.

**Table 1:** The number of genotyped and non-genotyped animals by generation in pedigree.

Generation	Genotyped				Non-genotyped	All
	Sib-tested	Parent	Unselected	All	Parent	
G0	0	0	0	0	46	46
G1	124	58	0	182	4	186
G2	135	53	0	188	2	190
G3	126	54	0	180	1	181
G4	119	49	9	177	4	181
G5	118	0	29	147	0	147
Total	622	214	38	874	57	931

A total of 22 traits were used in this study and are listed in Table 2. The details of the measurement methods are described by Kadowaki et al.^3^, Okamura et al.^5,10^, and Sato et al.^6^. In brief, average daily gain from birth to 105 kg BW (TDG) and from birth to 30 kg BW (DG30), DG105, and BF were measured for production traits. For respiratory disease traits, the MPS score were expressed as a scale of 0 to 100%^11^ according to the proportion of the lobe that formed hepatized lung tissue, which is a sign of Mhp infection. The AR score were expressed as a scale of 0 to 4^10^ according to the visually scored atrophic changes severity from cross section between the cuspid and first premolar in each snout. The pigs were infected with respiratory diseases under natural conditions. For immune-related traits, whole blood was collected from the cranial vena cava of the pigs under anesthesia at 7 weeks of age and 105 kg BW. Phagocytic activity at 105 kg BW (PA_105) and at 7 weeks (PA_7w) was determined in heparinized blood using chemiluminescence analysis. The total number of white blood cells at 105 kg BW (WBC_105) and 7 weeks off age (WBC_7w) was measured in ethylenediaminetetraacetic acid (EDTA)-treated whole blood. The ratio of granular leucocytes to lymphatic cells at 105 kg BW (RGL_105) and at 7 weeks of age (RGL_7w) in heparinized blood was measured. The complement alternative pathway activity in serum at 105 kg BW (CAPA_105) and at 7 weeks of age (CAPA_7w) was measured as the change in light-scattering properties of rabbit erythrocytes upon lysis. The CORT_105 and plasma concentration of cortisol at 7 weeks of age (CORT_7w) were also measured. Antibody production at 105 kg BW (AP) was determined by measuring the titer of IgG antibodies against sheep red blood cells (SRBC) after two inoculations with SRBC at 70 kg BW and at 100 kg BW. Cytokine concentrations of interleukin (IL)-10, IL-13, IL-17, interferon (IFN)-γ, and tumor necrosis factor (TNF)-α in the peripheral blood serum were measured in sib-tested pigs slaughtered at 105 kg BW.

**Table 2:** Descriptive statistics and estimated genetic parameter of the study subjects.

Traits^a^	Abbreviation	Unit^b^	Descriptive statistics			Estimated genetic parameter^c^			
			N	Mean	SD	Phenotypic variance		Heritability	
**Production traits**									
Average daily gain from birth to 105 kg body weight (BW)	TDG	g/day	857	650	71	2849	(179)	0.53	(0.07)
Average daily gain from birth to 30 kg BW	DG30	g/day	479	451	36	1118	(87)	0.21	(0.10)
Average daily gain from 30 to 105 kg BW	DG105	g/day	483	858	118	8649	(716)	0.53	(0.09)
Ultrasound backfat thickness	BF	mm	468	22.76	3.91	11.97	(1.02)	0.63	(0.08)
**Respiratory disease traits**									
Atrophic rhinitis score	AR score	–	618	0.93	0.81	0.64	(0.04)	0.28	(0.09)
Lesion score of mycoplasma pneumonia of swine	MPS score	%	620	−1.76	0.51	0.22	(0.01)	0.35	(0.10)
**Immune-related traits**									
Phagocytic activity at 105 kg BW	PA_105	10^6^ RLU	848	0.52	0.33	0.08	(0.00)	0.19	(0.06)
Phagocytic activity at 7-week old	PA_7w	10^6^ RLU	857	0.54	0.32	0.09	(0.01)	0.19	(0.09)
Complement alternative pathway activity at 105 kg BW	CAPA_105	OD_413_	846	−0.48	0.34	0.08	(0.00)	0.03	(0.03)
Complement alternative pathway activity at 7-week old	CAPA_7w	OD_413_	842	−0.50	0.29	0.07	(0.00)	0.09	(0.07)
Total number of white blood cells at 105 kg BW	WBC_105	×10^4^/mL	839	0.27	0.08	0.01	(0.00)	0.25	(0.07)
Total number of white blood cells at 7-week old	WBC_7w	×10^4^/mL	846	0.28	0.10	0.01	(0.00)	0.30	(0.10)
Ratio of granular leucocyte to lymph cells at 105 kg BW	RGL_105	–	850	−0.16	0.26	0.03	(0.00)	0.10	(0.05)
Ratio of granular leucocyte to lymph cells at 7-week old	RGL_7w	–	682	−0.17	0.19	0.03	(0.00)	0.03	(0.07)
Plasma concentrations of cortisol at 105 kg BW	CORT_105	µg/dL	764	−0.01	0.35	0.11	(0.01)	0.09	(0.05)
Plasma concentrations of cortisol at 7-week old	CORT_7w	µg/dL	468	0.32	0.30	0.08	(0.01)	0.21	(0.11)
Antibody production at 105 kg BW	AP	Titre	840	1.68	0.39	0.13	(0.01)	0.32	(0.07)
Serum concentration of interleukin 10 at 105 kg BW	IL-10	pg/mL	550	0.81	0.73	0.54	(0.03)	0.20	(0.09)
Serum concentration of interleukin 13 at 105 kg BW	IL-13	pg/mL	517	2.96	0.58	0.32	(0.02)	0.08	(0.07)
Serum concentration of interleukin 17 at 105 kg BW	IL-17	pg/mL	554	1.44	0.65	0.42	(0.03)	0.27	(0.10)
Serum concentration of interferon γ at 105 kg BW	IFN-γ	pg/mL	346	1.56	0.90	0.46	(0.04)	0.00	0.00
Serum concentration of tumor necrosis factor α at 105 kg BW	TNF-α	pg/mL	258	1.89	0.44	0.20	(0.02)	0.24	(0.16)

^a^MPS and immune-related traits were transformed to the natural logarithmic scale and the descriptive statistics of the transformed values are shown.

^b^Titer unit is equivalent to 104/dilution degrees of sample serum.

^c^Standard errors are shown in parentheses.

As the distributions of the phenotypic values were highly skewed^6,10^ for detecting the MPS score and immune-related traits, these phenotypic values were transformed to the natural logarithmic scale using the formula ${\mathrm{log}}_{\mathrm{e}}\left(\mathrm{x}\right)$ for immune-related traits and the formula ${\mathrm{log}}_{\mathrm{e}}\left(\frac{\mathrm{x}+0.5}{100-x+0.5}\right)$^12^ for the MPS score, where x is a phenotypic value. Phenotypic values within a mean ± 3 SD were used in this study and the descriptive statistics of these traits are shown in Table 2.

### SNP genotyping

Genomic DNA was extracted from ear tissue, as previously described by Okamura et al.^10^. Sample DNA was quantified and genotyped using the Illumina PorcineSNP60 BeadChip (v1 and v2; Illumina, San Diego, CA, USA) according to the manufacturer’s protocol. Image data were analyzed with the iScan (Illumina, San Diego, CA, USA) system and the genotype data were then called using the genotyping module contained in the GenomeStudio software (Illumina, San Diego, CA, USA). All SNP positions were updated according to the SNPchiMp v.3 database^13^ and the Sscrofa 11.1 reference sequence assembly downloaded from Ensembl (release 97) (http://ftp.ensembl.org/pub/release-97/variation/vcf/sus_scrofa/). SNP quality control was assessed using the PLINK 1.9 software^14^. The exclusion criteria for SNPs were minor allele frequency (MAF) < 0.05, call rate < 0.95, and Hardy–Weinberg equilibrium test with *p*-value < 0.001. The exclusion criterion for pigs was a call rate < 0.95. After quality control, a total of 874 pigs genotyped at 37,299 SNPs on autosomal *Sus scrofa* chromosomes (SSC) were available for GWAS.

### LD information

The LD coefficient (r^2^) values, which are a measure of LD, were calculated for all pairs of SNPs that were less than 10 Mbp apart using the PLINK 1.9 software^14^. Average r^2^ values for a given intermarker distance, with marker distances grouped in 2 kbp bins, were estimated for each autosome and the average r^2^ values among chromosomes were then calculated.

### SNP-based GWAS

We performed a SNP-based GWAS to detect significant SNPs. The adjusted phenotypes were first obtained using the single-trait animal model as follows:

1 $$\mathbf{y}=\mathbf{X}\mathbf{b}+\mathbf{Z}\mathbf{u}+\mathbf{W}\mathbf{c}+\mathbf{e},$$

where **y** is a vector of the observations; **X**, **Z**, and **W** are the known design matrices relating observations to fixed and random effects; **b** is a vector of fixed effects due to sex (three classes: boar, barrow, and gilt), the generation (five classes), and the rearing environment (three classes, only included in the traits at 105 kg BW); **u** is a vector of breeding values ($\mathbf{u}\sim N(0, \mathbf{A}{\upsigma }_{\mathrm{u}}^{2})$), where **A** and ${\upsigma }_{\mathrm{u}}^{2}$ are the additive relationship matrix and the additive genetic variance, respectively; **c** is a vector of common litter environmental effects (only included in the traits of 7-week-old-pigs and DG30) of dam ($\mathbf{c}\sim N\left(0, \mathbf{I}{\upsigma }_{\mathrm{c}}^{2}\right)$), where **I** and ${\upsigma }_{\mathrm{c}}^{2}$ are the identity matrix and the common litter environmental variance, respectively; and **e** is a vector of residual effects ($\mathbf{e}\sim N\left(0, \mathbf{I}{\upsigma }_{\mathrm{e}}^{2}\right)$), where ${\upsigma }_{\mathrm{e}}^{2}$ is the residual variance. The ASReml 4.1 software^15^ was used to estimate the genetic variance, phenotypic variance, and heritability, and the estimated values are shown in Table 2. The random effects were also predicted and the adjusted phenotypes (**y**_adj_) were then derived by

$${\mathbf{y}}_{\mathrm{adj}}=\widehat{\mathbf{u}}+\widehat{\mathbf{e}},$$

where $\widehat{\mathbf{u}}$ and $\widehat{\mathbf{e}}$ are the predicted values of the breeding value and the residual value obtained in model (1), respectively.

The adjusted phenotypes were used as the dependent traits in a linear mixed model approach for each SNP:

2 $${\mathbf{y}}_{\mathrm{adj}}={1}_{\mathrm{n}}\mu +{\upbeta }_{\mathrm{i}}{\mathbf{w}}_{\mathrm{i}}+\mathbf{a}+{\varvec{\upvarepsilon}},$$

where ${1}_{\mathrm{n}}$ is a vector of *n* ones; $\mu$ is the mean; β_i_ is the allele substitution effect at the *i*-th SNP; **w**_i_ is a vector of SNP genotypes (coded as 0, 1, and 2 for the homozygote, heterozygote, and the other homozygote, respectively) at the *i*th SNP; **a** is a vector of additive genetic effects ($\mathbf{a}\sim N(0, \mathbf{G}{\upsigma }_{\mathrm{a}}^{2})$), where **G** and ${\upsigma }_{\mathrm{a}}^{2}$ are the genomic relationship matrix (GRM) proposed by VanRaden^16^ and the SNP-based genetic variance, respectively; and ${\varvec{\upvarepsilon}}$ is a vector of residual effects (${\varvec{\upvarepsilon}}\sim N\left(0, \mathbf{I}{\upsigma }_{\upvarepsilon }^{2}\right)$), where ${\upsigma }_{\upvarepsilon }^{2}$ is the residual variance. The regression coefficient and *p*-values tested by the Wald test were obtained using the genome-wide mixed-model association (GEMMA) software^17^. The proportion of phenotypic variance explained by the *i*-th SNP effect was calculated using the formula^18^:

$${\mathrm{Proportion}}_{\mathrm{i}}=\frac{2{\mathrm{p}}_{\mathrm{i}}\left(1-{\mathrm{p}}_{\mathrm{i}}\right){\widehat{\upbeta }}_{\mathrm{i}}^{2}}{{\widehat{\upsigma }}_{\mathrm{p}}^{2}},$$

where p_i_ is the MAF of the *i*th SNP, ${\widehat{\upbeta }}_{\mathrm{i}}$ the estimated allele substitution effect of the *i*th SNP obtained in model (2), and ${\widehat{\upsigma }}_{\mathrm{p}}^{2}$ the estimated phenotypic variance obtained in model (1) (Table 2).

### Haplotype-based GWAS

Haplotype-based GWAS was performed on the basis of pedigree and LD information, and the details were shown^9,19^. Haplotypes were constructed using the hidden Markov model with DualPHASE^20^, which assumes the number of ancestral haplotype states (K = 20). The haplotype-based association analysis was then conducted using a linear mixed model using the GLASCOW software^21^. The adjusted phenotypes in SNP-based GWAS were used as the dependent traits in the model as follows:

$${\mathbf{y}}_{\mathrm{adj}}={1}_{\mathrm{n}}\mu +{\mathbf{H}}_{\mathrm{i}}{\mathbf{h}}_{\mathrm{i}}+{\mathbf{a}}_{\mathrm{H}}+{\varvec{\upvarepsilon}},$$

where **H**_i_ is the design matrix of haplotype genotypes for the individuals at the *i*-th haplotype locus relating observations to random effect; **h**_i_ is a vector of the haplotype effect (${\mathbf{h}}_{\mathrm{i}}\sim N(0, \mathbf{I}{\upsigma }_{\mathrm{h}}^{2})$), where ${\upsigma }_{\mathrm{h}}^{2}$ is haplotypic variance; and ${\mathbf{a}}_{\mathrm{H}}$ is a vector of additive genetic effects (${\mathbf{a}}_{\mathrm{H}}\sim N(0, {\mathbf{G}}_{\mathrm{H}}{\upsigma }_{{\mathrm{a}}_{\mathrm{H}}}^{2})$), where ${\mathbf{G}}_{\mathrm{H}}$ and ${\upsigma }_{{\mathrm{a}}_{\mathrm{H}}}^{2}$ are the haplotype-based GRM and the haplotype-based genetic variance, respectively^21,22^. After calculating the haplotype-based GRM, 0.00001 was added to diagonal elements to avoid near singularity problems. Using the GLASCOW software, associations were tested for every marker position along the genome by a significance test as follows: ${\upsigma }_{\mathrm{h}}^{2}=0$.

### Multi-trait meta-analysis

To evaluate the possibility of a pleiotropic QTL among traits, a multi-trait meta-analysis using the results of SNP-based GWAS was performed, and the approximate multi-trait test statistic described by Bolormaa et al.^23^ was calculated in R software (http://www.r-project.org) as follows:

$${\upchi }_{\mathrm{df}=22}^{2}={\mathbf{t}}_{\mathrm{i}}^{\mathbf{^{\prime}}}{\mathbf{V}}^{-1}{\mathbf{t}}_{\mathrm{i}},$$

where **t**_i_ is a vector of signed t-value at the *i*th SNP for the 22 traits, and the element of **t**_i_ is ${\upbeta }_{\mathrm{ij}}/se\left({\upbeta }_{\mathrm{ij}}\right)$ (${\upbeta }_{\mathrm{ij}}$ is the allele substitution effect at the *i*th SNP for the *j*-th trait and $se\left({\upbeta }_{\mathrm{ij}}\right)$ is the corresponding standard error); and ${\mathbf{V}}^{-1}$ is the inverse of the 22 × 22 correlation matrix between traits calculated from these signed t-values. These allele substitution effects and their standard errors were obtained from the results of 22 single-trait SNP-based GWAS.

### Gene and functional annotation of target SNPs

For the results of GWAS, Bonferroni correction was applied to determine the 5% genome-wide significance thresholds (*p-*value = 0.05/37,299 = 1.34 × 10^–6^). The extent of LD in this population was approximately 400 kbp (see Results), and the genome-wide significance thresholds defined by the Bonferroni correction were too conservative. Therefore, the genome-wide suggestive threshold^24^ was also defined as *p-*value = 1/37,299 = 2.68 × 10^–5^. The positional candidate genes within the range of the significant and suggestive SNPs ± 200 kbp region were annotated using the Ensembl database (release 97) (http://ftp.ensembl.org/pub/release-97/gff3/sus_scrofa/). To better understand the genes involved in biological processes, Gene Ontology (GO) and Kyoto Encyclopedia of Genes and Genomes (KEGG) pathways analyses were conducted based on genes within significant and suggestive SNPs ± 200 kbp region using the database for annotation, visualization, and integrated discovery (DAVID v6.8, https://david.ncifcrf.gov/). The significance of the enriched GO terms and KEGG pathways was assessed with *p*-value < 0.05 and at least three involved genes.

### Ethics statement

Animal Care and Use Committee approval was not obtained for this study because the phenotype and pedigree data were obtained from an existing database^3,5,6^ and DNA samples for DNA^10^ were used for SNP genotyping.

## Results

### LD in Landrace pigs

Average r^2^ values plotted against intermarker distance are shown in Supplementary Fig. S1 online. The results showed that moderate LD (r^2^ = 0.20) extended to about 400 kbp in this population.

### Single-trait GWAS

We performed SNP-based and haplotype-based GWAS for a total of 22 performance, respiratory disease, and immune-related traits in MPS-selected pigs. The values of an inflation factor were less than 1.1 in all results, and thus the results successfully accounted for population stratification.

**Figure 1:**
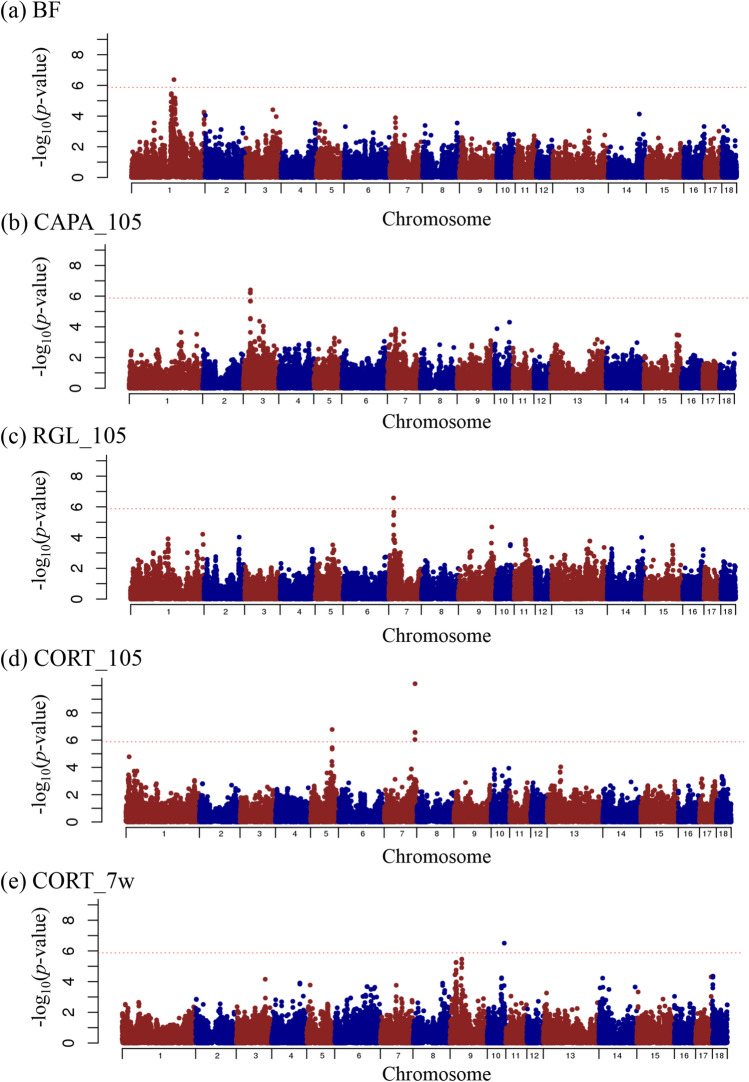
Manhattan plots representing the single nucleotide polymorphism (SNP)-based genome-wide significant association with production and immune-related traits in Landrace pigs. Abbreviations of the traits for BF **(a)**, CAPA_105 **(b)**, RGL_105 **(c)**, CORT_105 **(d),** and CORT_7w **(e)** are shown in Table 2. The x-axis indicates the chromosome number and the y-axis indicates –log_10_(*p*-value). The dotted horizontal line indicates the significant threshold.

For SNP-based GWAS, genome-wide plots of *p-*values with genome-wide significant SNPs and its details are shown in Fig. 1 and Table 3, respectively. Genome-wide plots of *p-*values with genome-wide suggestive SNPs and its details are also shown in Supplementary Fig. S1 online and Supplementary Table S1 online, respectively. A total of 11 genome-wide significant SNPs were detected in 5 traits and 59 genome-wide suggestive SNPs were detected in 14 traits. For production traits, a genome-wide significant SNP (rs80975749) for BF was detected on SSC1. No significant SNPs were detected in this study for respiratory disease traits. For immune-related traits, one genome-wide significant SNP was detected on SSC7 for RGL_105 (rs80902125) and on SSC10 for CORT_7w (rs81236875), and three SNPs for CAPA_105 (rs81229756, rs81312964, and rs81379304) were detected within a 60 kbp region of SSC3. Four SNPs (rs80996428, rs80918930, rs80966458, and rs80953170) within a 57 kbp region of SSC7 and one SNP (rs81326027) on SSC5 were significantly associated with CORT_105. The rs80918930 SNP at 116 Mbp on SSC7 had the highest significance in CORT_105 (*p-*value = 7.35 × 10^–11^, proportion = 0.06).

**Table 3:** The genome-wide significant single nucleotide polymorphisms (SNPs) associated with production and immune-related traits.

	SNP information					SNP effect				Gene symbol within the SNP ± 200 kbp region
Traits^a^	SSC	Position (bp)	refSNP variation ID	EA	EAF	Β^b^		Proportion^c^	*p*-value	
**Production traits**										
BF	1	########	rs80975749	A	0.46	−1.43	(0.28)	0.08	4.23E−07	SERPINB13,SERPINB12,SERPINB5,VPS4B,KDSR
**Immune-related traits**										
CAPA_105	3	2,64,34,892	rs81229756	A	0.09	−0.13	(0.03)	0.04	6.08E−07	TMC5,TMC7,COQ7,ITPRIPL2,SYT17
CAPA_105	3	2,64,39,289	rs81312964	A	0.09	−0.13	(0.03)	0.04	6.08E−07	TMC5,TMC7,COQ7,ITPRIPL2,SYT17
CAPA_105	3	2,70,30,167	rs81379304	A	0.09	−0.13	(0.03)	0.04	3.92E−07	XYLT1
RGL_105	7	1,82,10,546	rs80902125	G	0.12	−0.06	(0.01)	0.03	2.63E−07	–
CORT_105	5	8,08,08,190	rs81326027	G	0.48	0.10	(0.02)	0.04	1.67E−07	NT5DC3
CORT_105	7	########	rs80996428	A	0.27	0.10	(0.02)	0.04	9.14E−07	192405031432500PRIMA1,ASB2,CCDC197,OTUB2
CORT_105	7	########	rs80918930	C	0.40	0.11	(0.02)	0.06	7.35E−11	ISG12(A),PPP4R4,SERPINA6,SERPINA1,SERPINA11,UABP-2,SERPINA12
CORT_105	7	########	rs80966458	G	0.13	0.14	(0.03)	0.04	2.72E−07	PPP4R4,SERPINA6,SERPINA1,SERPINA11,UABP-2,SERPINA12
CORT_105	7	########	rs80953170	G	0.29	0.10	(0.02)	0.04	2.74E−07	PPP4R4,SERPINA6,SERPINA1,SERPINA11,UABP-2,SERPINA12,SERPINA4,SERPINA5
CORT_7w	10	6,34,10,258	rs81236875	A	0.05	0.19	(0.04)	0.04	3.12E−07	GATA3,TAF3,ATP5F1C,KIN

*SSC* *Sus scrofa* chromosome, *EA* effect allele, *EAF* effect allele frequency.

^a^Abbreviations of traits are shown in Table 2.

^b^Standard errors are shown in parentheses.

^c^The proportion of adjusted phenotypic variance explained by the SNP effects.

**Figure 2:**
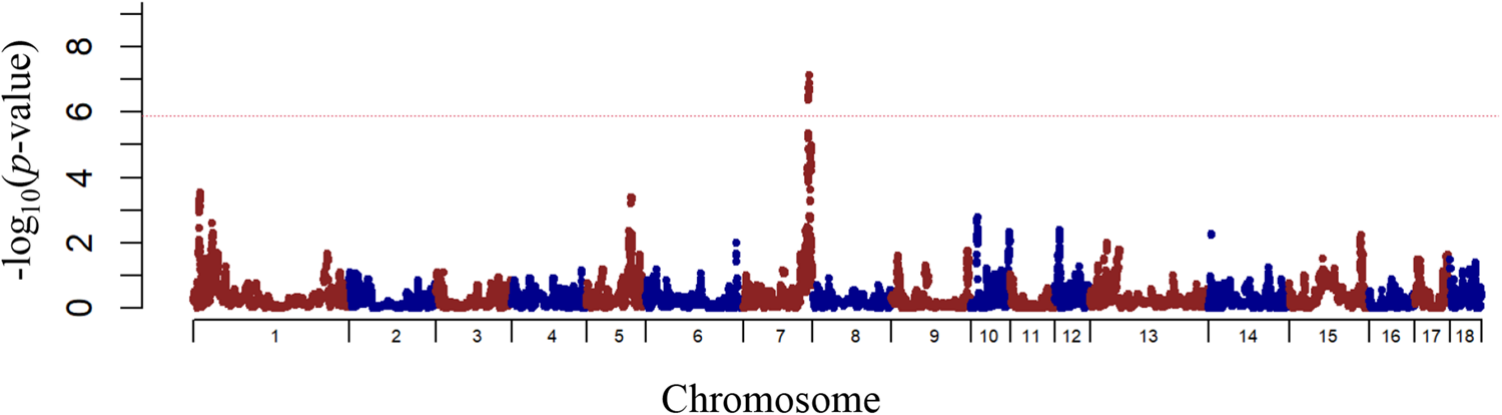
Manhattan plot representing the haplotype-based genome-wide significant association with cortisol at 105 kg body weight in Landrace pigs. The x-axis indicates the chromosome number and the y-axis indicates –log_10_(*p*-value). The dotted horizontal line indicates the significant threshold.

For haplotype-based GWAS, genome-wide plots of *p-*values with genome-wide significant SNPs and its details are shown in Fig. 2 and Supplementary Table S2 online, respectively. Genome-wide plots of *p-*values with genome-wide suggestive SNPs and its details are also shown in Supplementary Fig. S3 online and Supplementary Table S2 online, respectively. Only one genome-wide significant region on SSC7 for CORT_105 was detected, and one genome-wide suggestive region on SSC12 for IFN-γ was detected. The significant region at 115–116 Mbp on SSC7 for CORT_105 and the suggestive region at 14–15 Mbp on SSC12 for IFN-γ were overlapped across the regions detected by SNP-based GWAS.

For gene and functional annotation analyses, several candidate genes within the range of the significant and suggestive SNPs ± 200 kbp regions are shown in Table 3 and Supplementary Table S1 online for SNP-based GWAS, and in Supplementary Table S2 online for haplotype-based GWAS. The lists of these candidate genes for each trait were assembled into GO and KEGG pathways analyses using DAVID database, and the results are shown in Supplementary Table S3 online. The significant GO terms were detected in seven traits (TDG, BF, WBC_105, RGL_105, CORT_105, IFN-γ, and TNF-α) and the significant KEGG pathways were detected in one trait (IFN-γ). The most significant GO term was related to serine-type endopeptidase inhibitor activity (GO:0004867) in CORT_105 (*p-*value = 7.00 × 10^–9^).

### Multi-trait meta-analysis and overlapping regions across studies

A multi-trait meta-analysis was performed for the 22 traits using the results of SNP-based GWAS to evaluate the presence of a pleiotropic QTL among traits. The multi-trait meta-analysis detected no genome-wide significant region and six genome-wide suggestive regions were detected on SSC2, 3, 4, 7, and 10 (Supplementary Table S1 online). Only two of the six regions were overlapped across multiple regions detected by single-trait GWAS, and there were the regions on SSC2 (for WBC_105 and WBC_7w) and SSC7 (for MPS score and CORT_105). For gene and functional annotation analyses, several candidate genes within the range of the suggestive SNPs ± 200 kbp regions are shown in Supplementary Table S1 online, and the results of GO and KEGG pathways analyses are shown in Supplementary Table S3 online. Three significant GO terms were detected, and the most significant GO term (GO:0004867) in CORT_105 was included in the terms.

**Figure 3:**
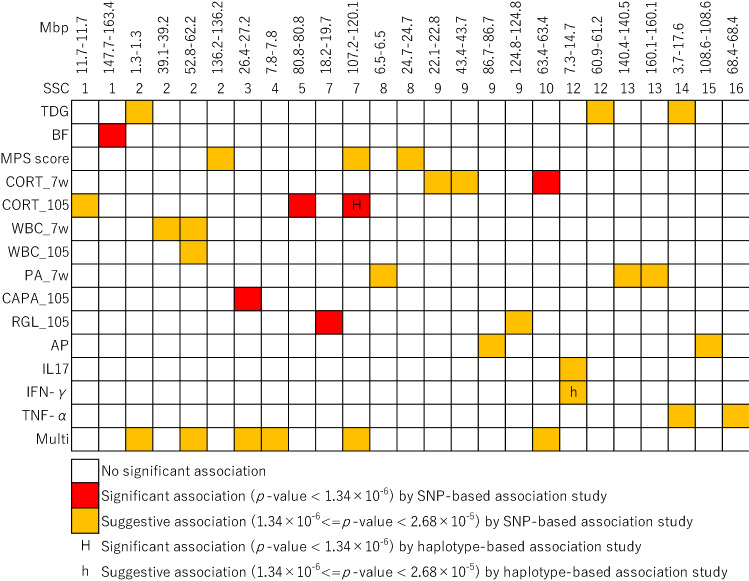
The summary regions associated with traits across genome. Each row represents the results of single nucleotide polymorphisms (SNPs)-based and haplotype-based genome-wide association studies (GWAS) in each trait. Each column represents a genomic region containing SNPs with suggestive and significant levels in each result. For the results of GWAS, and *p-*value = 2.68 × 10^–5^ and *p-*value = 1.34 × 10^–6^ were regarded as genome-wide suggestive and significant associations with a trait, respectively. Only traits with at least one associated SNP and SNPs associated with at least one trait are shown. Abbreviations of the traits are shown in Table 2.

We summarized the regions associated with traits across the genome in Fig. 3. With regard to the pleiotropic QTL among traits, only three of these genome-wide significant and suggestive regions overlapped among traits (the region on SSC2 for WBC_105 and WBC_7w; the region on SSC7 for MPS score and CORT_105; and the region on SSC12 for IL17 and IFN-γ), and two of the three overlapped regions were overlapped across the regions detected by multi-trait meta-analysis.

## Discussion

### QTL detection for production, respiratory disease, and immune-related traits

It is important to understand the genomic background of the relationship among production, respiratory disease, and immune-related traits by detecting the QTL for these traits to increase animal productivity through genetic selection for disease resistance. Several GWAS studies have reported significant QTLs for immune-related traits such as hematological^25–27^, T lymphocyte subpopulations^28,29^, and cytokine levels^30,31^ in different purebred and crossbred pigs. However, there are few genomic regions that overlap among reports, and this strongly depends on the genetic background of the studies. Only a few studies have reported GWAS in respiratory disease^32^, and the details of the genomic relationship among production, respiratory disease, and immune-related traits have not yet been reported. Therefore, we performed GWAS for a total of 22 performance, respiratory disease, and immune-related traits in MPS-selected pigs.

SNP-based GWAS detected a total of six genome-wide significant regions in five traits and some of the genome-wide suggestive regions in 14 traits. For the results of relationships among traits, only three regions were associated with multiple traits, and most of the regions did not overlap among traits. In addition, a multi-trait meta-analysis was performed to evaluate the possibility of a pleiotropic QTL among these traits, and no genome-wide significant region was detected. For genetic correlation among production, respiratory disease, and immune-related traits, Clapperton et al.^33^ reported that several of the peripheral blood mononuclear leukocyte subsets were negatively genetically correlated with daily gain in Large White pigs. Flori et al.^34^ also reported that genetic correlations among immune-related traits were weak, except for a few traits that mostly include cell subsets in Large White pigs. In our population, there were low genetic correlations between production and respiratory disease traits^3,5^, low-to-moderate genetic correlations between production and immune-related traits^5,6^, and low-to-moderate genetic correlations among immune-related traits^5,6^. As a result of the detected QTL in this study, many regions independently affected the traits. Thus, a polygenic effect could contribute to the genetic correlation among the traits when the genetic correlations between traits are moderate.

Okamura et al.^10^ performed linkage-based QTL mapping using microsatellite markers to detect a QTL for respiratory disease and immune-capacity traits in the same population as our study. They detected a significant QTL for WBC_7w and WBC_105 in the region on SSC2, which was the same region as that detected in our study. They also detected a significant QTL for CORT_105 on SSC7 with the highest significance, on which the most significant SNPs were also detected in our study. Although some of these regions were similar to those reported by Okamura et al.^10^, novel QTLs were detected in several genomic regions in our study. The population used in this study was closed-line breeding population, and the selection for MPS resistance was conducted at one generation per year to be identical the performance test environment at each selection generation^3^. Thus, the environmental condition can be controlled and adjusted to perform statistical analysis. In addition, the power of SNP-based GWAS was greater than that of haplotype-based GWAS in a Duroc multigenerational population^9^, which is similar pedigree structure in our population, and a novel QTL could be detected by SNP-based GWAS as opposed to linkage-based GWAS. Therefore, we think that the novel significant QTLs were detected in our population, even if most of immune-related traits had low heritability.

As for MPS score, a significant QTL was not detected in our study, whereas a significant QTL for MPS score was detected in the upper region on SSC2 by linkage-based QTL mapping using microsatellite markers^10^. Linkage-based QTL mapping considers only the association between DNA markers and QTL in the larger LD region, which is defined only by within-family recombination. In addition, because of the low density of microsatellite markers, these QTL are generally located with poor accuracy. In this study, we performed haplotype-based GWAS accounted for within-family recombination using high-density SNP array, but only one significant region was detected. The detected significant region for CORT_105 on SSC7 had the highest significance in the result of Okamura et al.^10^. In addition, the significant and suggestive regions were overlapped across the regions detected by SNP-based GWAS. The SNP-based GWAS using high-density SNP array consider the association in the LD region across the entire population^35^. It is considered that the frequency of a specific haplotype, which is not related to the MPS score and is the extent for longer distance, changed from G0 to G5 by selection for MPS resistance, and thus, the detected QTL for MPS score by linkage-based QTL mapping may show false positives.

### Candidate genes in the detected regions

GWAS detected a total of six significant QTL in this study. The significant QTL for CORT_105 was detected on SSC7, which was previously reported in different breeds^36–38^. The *corticosteroid-binding globulin* (*CBG*, also known as *SERPINA6*) gene was regarded as a positional candidate gene in the QTL region. The *SERPINA6* gene encodes CBG affecting cortisol-binding capacity, and some GO terms were significantly associated with *SERPINA6* gene (Supplementary Table S3 online). SNPs at the *SERPINA6* locus influence plasma cortisol levels in humans^39^ and pigs^36–38^. Our results supported the finding that the *SERPINA6* gene would be a positional candidate gene for CORT_105. The other regions detected in our study were not detected by previous linkage-based QTL mapping and were novel regions. No candidate gene was located on the significant QTL for RGL_105. However, some candidate genes were located in the QTL regions.

For production traits, the significant QTL for BF was detected on SSC1, and *vacuolar protein sorting-associated protein 4B* (*VPS4B*) gene was the candidate gene in the QTL region. The SNPs near the *VPS4B* gene were associated with abdominal fat in chicken, and a significantly different expression of the *VPS4B* gene was reported in cohorts of chickens with the highest and lowest abdominal fat content^40^.

For immune-related traits, the significant QTL for CAPA_105 was detected on SSC3 and the *Synaptotagmin-17* (*SYT17*) gene was the candidate gene in the QTL region. SYT17 is increased in the exosomal fraction of urine, and this increase is associated with activation of the IL-6 amplifier in humans^41^. Complement component 3 (C3), which plays a vital role in CAPA, is an acute-phase protein whose expression is regulated by cytokines such as IL-6^42^. Thus, the *SYT17* gene could be indirectly associated with CAPA_105. The significant QTL for CORT_105 was detected on SSC5, and the *5'-Nucleotidase Domain Containing 3* (*NT5DC3*) gene was the candidate gene in the QTL regions. NT5DC3 is a mitochondrion-related protein, and a difference in gene expression between high- and low-stress reactivity in mouse lines was observed in the *NT5DC3* gene^43^. The significant additive QTL for CORT_7w was detected on SSC10 and the *GATA binding protein 3 (GATA3)* gene was the candidate gene in the QTL region. GATA3 is upregulated during T helper (Th)2 cell differentiation and glucocorticoids such as cortisol increase Th2 activity^44^, and thus the expression of *GATA3* gene might indirectly affect CORT_7w.

Three QTL regions detected by single-trait GWAS were overlapped among traits, and there were the regions on SSC2 (for WBC_105 and WBC_7w), SSC7 (for MPS score and CORT_105), and SSC12 (for IL17 and IFN-γ). The candidate gene on SSC7 would be *SERPINA6* gene as shown above, and some candidate genes were located in the QTL regions on SSC2 and SSC12. The QTL for WBC_105 and WBC_7w was detected on SSC2, and *RAS protein activator like 3 (RASAL3)* gene was the candidate gene in the QTL region. RASAL3 regulates the number and functions of natural killer T cells^45^. RASAL3 is significantly upregulated in *Mycoplasma pneumoniae* pneumonia (MPP) children, and NK cells are involved in the pathogenesis of MPP^46^. The QTL for IL17 and IFN-γ was detected on SSC12, *calcium ion channel gamma subunit (CACNG)* gene cluster (*CACNG1*, *CACNG4*, *CACNG5*) was the candidate genes in the QTL region. The *CACNG* genes encode the gamma subunits of a voltage-dependent calcium channels, and some GO terms and KEGG pathways were significantly associated with *CACNG* gene cluster (Supplementary Table S3 online). the SNP located in the region of *CACNG4* and *CACNG5* gene was significantly associated with schizophrenia in human^47^. Schizophrenia has been consistently linked to chronic low-grade inflammation, and Th1/Th2 cytokine balance and activation of Th17 pathway play an important role in schizophrenia^48^. Th1-type cytokines such as IFN-γ is one of potent inhibitors of Th17 development, and Th17 produce IL-17. Thus, the function of *CACNG* genes might indirectly affect IL17 and IFN-γ. From these reasons, these candidate genes could be associated with the traits, and further study is needed to understand the details of genomic mechanisms.

## Conclusion

In this study, we performed GWAS for a total of 22 production, respiratory disease, and immune-related traits in MPS-selected pigs. Our GWAS results showed a total of six significant QTLs for these traits. The detected regions except for the region on SSC7 for CORT_105 were not detected by previous linkage-based QTL mapping, and these regions were novel regions with some candidate genes. With regard to a pleiotropic QTL among traits, only a few detected QTL regions overlapped among traits. A few genome-wide significant and suggestive regions were detected by haplotype-based GWAS, and the detected regions were overlapped across the regions detected by SNP-based GWAS. The study provided new insights into the genomic factors affecting production, respiratory disease, and immune-related traits in pigs.

## Supplementary Information

Supplementary Figure S1.

Supplementary Figure S2.

Supplementary Figure S3.

Supplementary Table S1.

Supplementary Table S2.

Supplementary Table S3.

## Data Availability

All information supporting the conclusions of this article are included within the article and its additional files. The raw datasets used in the current study are available from the authors upon reasonable request and with permission of Miyagi Prefectural Animal Industry Experiment Station.
