# Supplementary material for: Genome-wide association studies for production, respiratory disease, and immune-related traits in Landrace pigs
Source: Sci Rep. 2021 Aug 4;11:15823. doi: 10.1038/s41598-021-95339-2 (PMC8338966; doi:10.1038/s41598-021-95339-2)
Supplement: Supplementary file 2 — Supplementary Figure S2. [file 41598_2021_95339_MOESM2_ESM.pdf]

Genome-wide association studies for production, respiratory disease, and immune-related traits in Landrace pigs

Yoshinobu Uemoto[1†*], Kasumi Ichinoseki[1†], Toshimi Matsumoto[2], Nozomi Oka[3], Hironori Takamori[3], Hiroshi Kadowaki[3], Chihiro Kojima-Shibata[3], Eisaku Suzuki[3], Toshihiro Okamura[4], Hisashi Aso[1], Haruki Kitazawa[1], Masahiro Satoh[1], Hirohide Uenishi[2], Keiichi Suzuki[1]

[1]Graduate School of Agricultural Science, Tohoku University, Sendai, Miyagi 980-8572, Japan.
[2]Animal Bioregulation Unit, Division of Animal Sciences, Institute of Agrobiological Sciences, National Agriculture and Food Research Organization (NARO), Tsukuba, Ibaraki 305-8634, Japan
[3]Miyagi Prefecture Animal Industry Experiment Station, Osaki, Miyagi 989-6445, Japan
[4]Institute of Livestock and Grassland Science, NARO, Tsukuba, Ibaraki 305-0901, Japan

[†]These authors have contributed equally to this work and share first authorship.
[*]Correspondence author

Supplementary Figure
Figure S2. Manhattan plots representing the single nucleotide polymorphism (SNP)-based genome-wide suggestive association with production, respiratory disease, and immune-related traits in Landrace pigs.

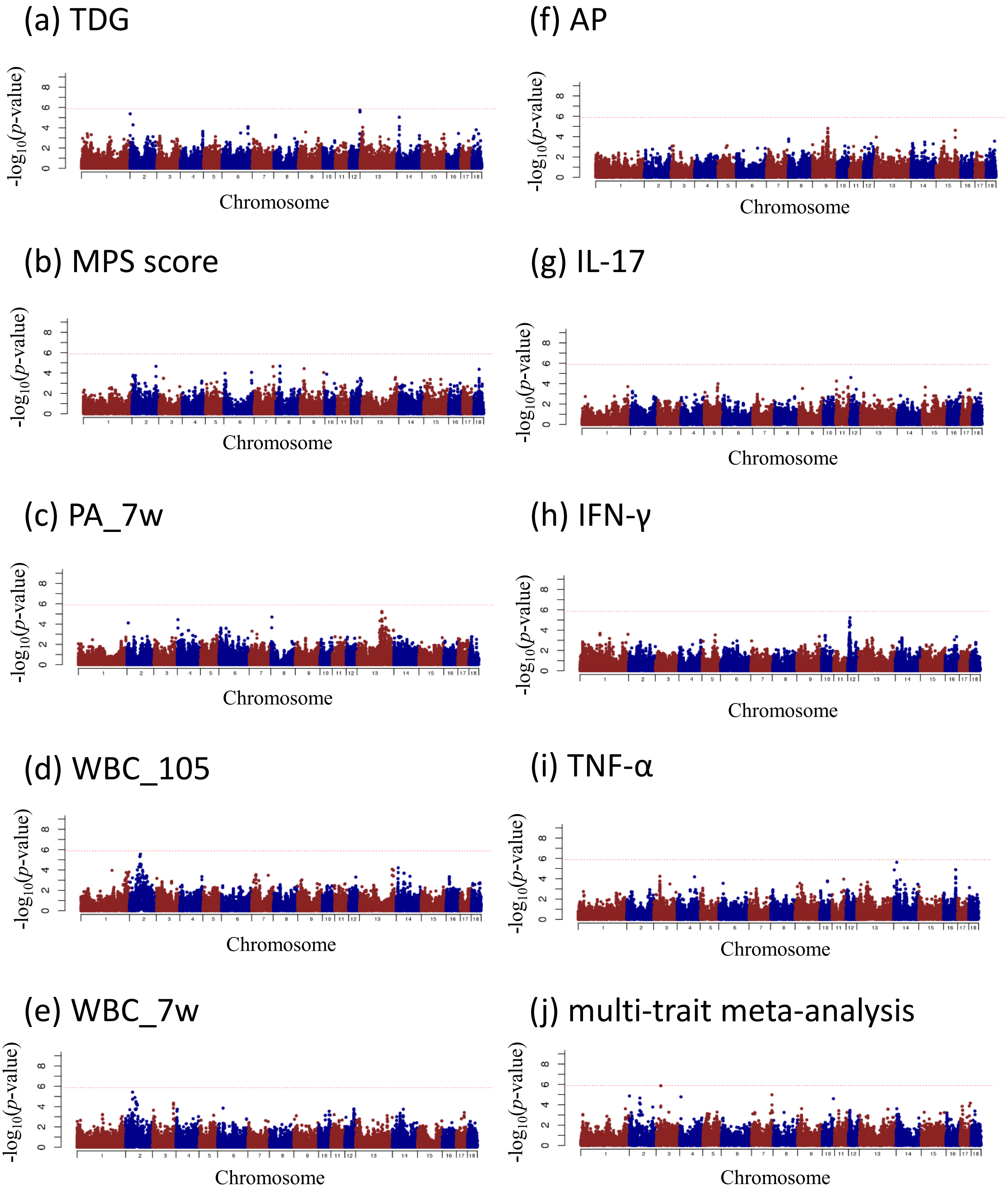

Figure S2. Manhattan plots representing the single nucleotide polymorphism (SNP)-based genome-wide suggestive association with production, respiratory disease, and immune-related traits in Landrace pigs. Abbreviations of the traits are shown in Table 2. The x-axis indicates the chromosome number and the y-axis indicates $-\log_{10}(p\text{-value})$. The dotted horizontal line indicates the significant threshold.
