# Supplementary material for: Genome-wide association studies for production, respiratory disease, and immune-related traits in Landrace pigs
Source: Sci Rep. 2021 Aug 4;11:15823. doi: 10.1038/s41598-021-95339-2 (PMC8338966; doi:10.1038/s41598-021-95339-2)
Supplement: Supplementary file 4 — Supplementary Table S1. [file 41598_2021_95339_MOESM4_ESM.pdf]

Genome-wide association studies for production, respiratory disease, and immune-related traits in Landrace pigs

Yoshinobu Uemoto[1†*], Kasumi Ichinoseki[1†], Toshimi Matsumoto[2], Nozomi Oka[3], Hironori Takamori[3], Hiroshi Kadowaki[3], Chihiro Kojima-Shibata[3], Eisaku Suzuki[3], Toshihiro Okamura[4], Hisashi Aso[1], Haruki Kitazawa[1], Masahiro Satoh[1], Hirohide Uenishi[2], Keiichi Suzuki[1]

[1]Graduate School of Agricultural Science, Tohoku University, Sendai, Miyagi 980-8572, Japan.
[2]Animal Bioregulation Unit, Division of Animal Sciences, Institute of Agrobiological Sciences, National Agriculture and Food Research Organization (NARO), Tsukuba, Ibaraki 305-8634, Japan
[3]Miyagi Prefecture Animal Industry Experiment Station, Osaki, Miyagi 989-6445, Japan
[4]Institute of Livestock and Grassland Science, NARO, Tsukuba, Ibaraki 305-0901, Japan

[†]These authors have contributed equally to this work and share first authorship.
[*]Correspondence author

Supplementary table

Table S1. The genome-wide suggestive single nucleotide polymorphisms (SNPs) associated with production, respiratory disease, and immune-related traits.

Table S1. The genome-wide suggestive single nucleotide polymorphisms (SNPs) associated with production, respiratory disease, and immune-related traits

Traits[a]	SNP information[b]					SNP effect			Gene symbol within the SNP ±200 kbp region
	SSC	Position(bp)	refSNP variation ID	EA	EAF	β[c]	Proportion[d]	*p*-value	
Production traits									
TDG	2	1,267,622	rs81243163	G	0.19	17.04 (3.68)	0.03	4.27E-06	IFITM10,CTSD,SYT8,TNNI2,LSP1,PRR33,TNNT3
	12	60,871,978	rs81288566	A	0.06	-27.89 (5.81)	0.03	1.87E-06	TOM1L2,SREBF1,RAI1,PEMT,RASD1,MED9,NT5M,COPS3,FLCN,PLD6
	12	61,215,277	rs81255656	A	0.06	-27.28 (5.78)	0.03	2.78E-06	COPS3,FLCN,PLD6,MPRIP,TNFRSF13B,USP22,DHRS7B,TMEM11,NATD1,MAP2K3
	14	13,891,794	rs80983361	A	0.06	-26.92 (6.04)	0.03	9.34E-06	MSRA,PRSS55,C8orf74,SOX7,XKR6
BF	1	147,654,193	rs81240401	A	0.45	1.21 (0.27)	0.06	1.20E-05	GALR1,MBP,ZNF236
	1	148,327,912	rs81349009	G	0.47	1.27 (0.27)	0.07	4.15E-06	-
	1	148,387,133	rs81251179	A	0.47	1.27 (0.27)	0.07	4.15E-06	-
	1	148,497,978	rs81349012	A	0.22	-1.56 (0.37)	0.07	2.56E-05	-
	1	148,839,311	rs81349042	A	0.31	1.38 (0.29)	0.07	3.43E-06	TSHZ1,ZADH2
	1	155,492,432	rs80986448	G	0.21	-1.71 (0.38)	0.08	7.30E-06	CDH19
	1	157,498,487	rs81349201	C	0.49	-1.22 (0.28)	0.06	1.41E-05	-
	1	162,105,226	rs80991377	C	0.44	-1.30 (0.29)	0.07	1.05E-05	ZNF532,MALT1,ALPK2
	1	162,147,201	rs80978205	G	0.49	-1.21 (0.28)	0.06	1.32E-05	ZNF532,MALT1,ALPK2
	1	162,192,627	rs81349302	G	0.50	-1.25 (0.27)	0.06	6.60E-06	MALT1,ALPK2,NEDD4L
	1	162,372,950	rs80896334	A	0.48	-1.26 (0.29)	0.07	1.28E-05	ALPK2,NEDD4L
	1	163,382,446	rs80864027	A	0.22	-1.75 (0.40)	0.09	1.69E-05	CLPX,CILP,PARP16,IGDCC3,IGDCC4,DPP8,HACD3,INTS14
Respiratory disease traits						0.00			
MPS score	2	136,232,960	rs81222517	A	0.45	-0.13 (0.03)	0.04	2.18E-05	C5orf15,TCF7
	7	107,229,684	rs336042505	A	0.44	0.13 (0.03)	0.04	2.30E-05	-
	8	24,735,805	rs81398421	C	0.06	-0.25 (0.06)	0.03	2.06E-05	-
Immune-related traits						0.00			
PA_7w	8	6,537,938	rs81343316	A	0.46	0.05 (0.01)	0.01	2.03E-05	SLC2A9,WDR1,ZNF518B,CLNK
	13	140,375,976	rs322548798	G	0.31	0.06 (0.01)	0.02	5.76E-06	GSK3B,NR1I2
	13	140,376,953	rs331134940	G	0.31	0.06 (0.01)	0.02	7.31E-06	GSK3B,NR1I2
	13	140,411,493	rs81448510	A	0.31	0.06 (0.01)	0.02	7.31E-06	GSK3B,NR1I2,COX17,POPDC2
	13	140,452,426	rs80842494	A	0.31	0.06 (0.01)	0.02	7.31E-06	GSK3B,NR1I2,COX17,POPDC2,PLA1A
	13	140,474,261	rs80796660	C	0.31	0.06 (0.01)	0.02	7.31E-06	GSK3B,NR1I2,COX17,POPDC2,PLA1A
	13	160,080,510	rs80802927	A	0.17	0.07 (0.02)	0.01	2.61E-05	DCBLD2,ST3GAL6
CAPA_105	3	26,483,338	rs81475132	C	0.06	-0.15 (0.03)	0.03	2.11E-06	TMC5,TMC7,COQ7,ITPRIPL2,SYT17,SMG1
	3	26,981,476	rs81475348	A	0.06	-0.15 (0.03)	0.03	2.11E-06	XYLT1
	3	27,180,639	rs81243013	A	0.06	-0.15 (0.03)	0.03	2.11E-06	XYLT1
WBC_105	2	58,888,838	rs81487680	G	0.30	-0.02 (0.01)	0.04	4.75E-06	MEF2B,TMEM161A,ARMC6,SUGP2,DDX49,UPF1,COMP,CRTC1
	2	59,738,035	rs322217417	A	0.29	-0.02 (0.01)	0.04	2.53E-05	RAB3A,MPV17L2,IFI30,MAST3,IL12RB1,ARRDC2,KCNN1,CCDC124,SLC5A5,JAK3,INSL3,B3GNT3
	2	60,108,314	rs81359730	G	0.30	-0.02 (0.01)	0.04	4.46E-06	JAK3,INSL3,B3GNT3,FCHO1,MAP1S,COLGALT1,FAM129C,SLC27A1,NXNL1,MVB12A,BST2
	2	62,246,385	rs80992257	G	0.30	-0.02 (0.01)	0.04	2.79E-06	CYP4F22,PGLYRP2,RASAL3,AKAP8L,AKAP8,BRD4,EPHX3,NOTCH3,ILVBL,SYDE1
WBC_7w	2	39,149,929	rs81357548	A	0.45	0.02 (0.01)	0.03	3.80E-06	PRMT3,HTATIP2,DBX1,NAV2
	2	39,217,661	rs81357600	A	0.42	0.02 (0.01)	0.02	1.87E-05	PRMT3,HTATIP2,DBX1,NAV2
	2	52,791,773	rs81237218	G	0.13	-0.03 (0.01)	0.02	1.27E-05	-
RGL_105	7	18,172,398	rs80807040	G	0.06	-0.08 (0.02)	0.02	1.52E-05	-
	7	18,645,244	rs80981748	A	0.10	-0.06 (0.01)	0.02	3.58E-06	-
	7	19,658,767	rs80932009	A	0.21	-0.05 (0.01)	0.03	2.23E-06	TDP2,ACOT13,C6orf62,GMNN,ARMH2
	9	124,790,147	rs81417044	A	0.37	0.04 (0.01)	0.03	2.03E-05	NMNAT2,SMG7,NCF2,ARPC5,RGL1,APOBEC4
CORT_105	1	11,684,450	rs80923830	A	0.39	0.08 (0.02)	0.03	1.67E-05	NOX3,TFB1M,CLDN20,TIAM2
	5	80,818,426	rs81245792	G	0.34	0.09 (0.02)	0.03	3.58E-06	NT5DC3
	5	80,820,281	rs81339682	G	0.34	0.09 (0.02)	0.03	4.58E-06	NT5DC3
CORT_7w	9	22,071,228	rs81343906	G	0.27	0.09 (0.02)	0.04	5.69E-06	GRM5
	9	22,573,159	rs81336772	G	0.48	0.07 (0.02)	0.03	1.76E-05	GRM5,TYR,NOX4
	9	22,763,662	rs81321075	G	0.23	0.09 (0.02)	0.03	5.53E-06	TYR,NOX4,FOLH1B
	9	43,420,493	rs81410136	G	0.16	-0.10 (0.02)	0.03	1.22E-05	-
	9	43,705,584	rs81410215	G	0.16	-0.10 (0.02)	0.03	6.48E-06	-
	9	43,739,452	rs81410233	A	0.17	-0.10 (0.02)	0.04	3.42E-06	-
AP	9	86,743,853	rs341975939	G	0.28	0.10 (0.02)	0.03	1.54E-05	AHR
	15	108,645,898	rs80990650	G	0.25	0.10 (0.02)	0.03	2.37E-05	PARD3B
IL-17	12	7,283,888	rs81437363	G	0.17	-0.24 (0.06)	0.04	2.49E-05	-
IFN-γ	12	10,974,664	rs81439586	A	0.50	0.22 (0.05)	0.05	2.09E-05	MAP2K6,ABCA5
	12	11,004,527	rs81439595	G	0.40	-0.22 (0.05)	0.05	1.31E-05	MAP2K6,ABCA5
	12	13,502,838	rs81440208	A	0.25	0.28 (0.06)	0.07	5.96E-06	CACNG5,CACNG4,CACNG1,HELZ
TNF-α	14	3,711,824	rs81327634	G	0.50	-0.17 (0.04)	0.08	1.35E-05	-
	14	17,600,242	rs341122035	C	0.47	-0.19 (0.04)	0.09	2.40E-06	-
	16	68,422,227	rs81461701	G	0.17	0.22 (0.05)	0.07	1.27E-05	MRPL22,GEMIN5,CNOT8,FAXDC2,LARP1
	16	68,425,822	rs81347008	A	0.17	0.22 (0.05)	0.07	1.27E-05	MRPL22,GEMIN5,CNOT8,FAXDC2,LARP1
Multi-trait meta-analysis									
	2	1,267,622	rs81243163					1.37E-05	IFITM10,CTSD,SYT8,TNNI2,LSP1,PRR33,TNNT3
	2	60,108,314	rs81359730					2.16E-05	JAK3,INSL3,B3GNT3,FCHO1,MAP1S,COLGALT1,FAM129C,SLC27A1,NXNL1,MVB12A,BST2
	3	26,483,338	rs81475132					1.36E-06	TMC5,TMC7,COQ7,ITPRIPL2,SYT17,SMG1
	3	26,981,476	rs81475348					1.36E-06	XYLT1
	3	27,180,639	rs81243013					1.36E-06	XYLT1
	4	7,783,321	rs80920273					1.65E-05	ST3GAL1
	7	115,550,783	rs80918930					1.04E-05	ISG12(A),PPP4R4,SERPINA6,SERPINA1,SERPINA11,UABP-2,SERPINA12
	10	63,410,258	rs81236875					2.53E-05	GATA3,TAF3,ATP5F1C,KIN

[a]Abbreviations of traits are shown in Table 2.

[b]SSC: Sus Scrofa chromosome, EA: Effect allele, EAF: Effect allele frequency.

[c]Standard errors are shown in parentheses.

[d]The proportion of adjusted phenotypic variance explained by the SNP efects.
