# Supplementary material for: Genome-wide association studies for production, respiratory disease, and immune-related traits in Landrace pigs
Source: Sci Rep. 2021 Aug 4;11:15823. doi: 10.1038/s41598-021-95339-2 (PMC8338966; doi:10.1038/s41598-021-95339-2)
Supplement: Supplementary file 5 — Supplementary Table S2. [file 41598_2021_95339_MOESM5_ESM.pdf]

Genome-wide association studies for production, respiratory disease, and immune-related traits in Landrace pigs

Yoshinobu Uemoto[1†*], Kasumi Ichinoseki[1†], Toshimi Matsumoto[2], Nozomi Oka[3], Hironori Takamori[3], Hiroshi Kadowaki[3], Chihiro Kojima-Shibata[3], Eisaku Suzuki[3], Toshihiro Okamura[4], Hisashi Aso[1], Haruki Kitazawa[1], Masahiro Satoh[1], Hirohide Uenishi[2], Keiichi Suzuki[1]

[1]Graduate School of Agricultural Science, Tohoku University, Sendai, Miyagi 980-8572, Japan.
[2]Animal Bioregulation Unit, Division of Animal Sciences, Institute of Agrobiological Sciences, National Agriculture and Food Research Organization (NARO), Tsukuba, Ibaraki 305-8634, Japan
[3]Miyagi Prefecture Animal Industry Experiment Station, Osaki, Miyagi 989-6445, Japan
[4]Institute of Livestock and Grassland Science, NARO, Tsukuba, Ibaraki 305-0901, Japan

[†]These authors have contributed equally to this work and share first authorship.
[*]Correspondence author

Supplementary table
Table S2. The genome-wide suggestive and significant single nucleotide polymorphisms (SNPs) associated with immune-related traits for haplotype-based association study.

Table S2. The genome-wide suggestive and significant single nucleotide polymorphisms (SNPs) associated with immune-related traits for haplotype-based association study

Traits[a]	SSC[b]	Position(bp)	refSNP varidation ID	*p*-value[c]	Gene symbol within the SNP ±200 kbp region
CORT_105	7	114,409,265	rs80859105	4.49E-06 *	RIN3,LGMN,GOLGA5,CHGA,ITPK1,TMEM251,GON7,UBR7
	7	114,460,289	rs80843547	1.37E-05 *	RIN3,GOLGA5,CHGA,ITPK1,TMEM251,GON7,UBR7,BTBD7
	7	114,480,337	rs80803653	1.37E-05 *	RIN3,CHGA,ITPK1,TMEM251,GON7,UBR7,BTBD7
	7	114,517,322	rs80792520	1.37E-05 *	RIN3,CHGA,ITPK1,TMEM251,GON7,UBR7,BTBD7
	7	114,585,094	rs80971641	6.52E-06 *	RIN3,ITPK1,TMEM251,GON7,UBR7,BTBD7
	7	114,607,342	rs80840002	6.52E-06 *	RIN3,ITPK1,TMEM251,GON7,UBR7,BTBD7
	7	114,674,294	rs341043600	5.64E-06 *	RIN3,ITPK1,TMEM251,GON7,UBR7,BTBD7
	7	114,999,265	rs80889242	5.87E-06 *	PRIMA1
	7	115,347,654	rs330303348	3.27E-07 **	ASB2,CCDC197,OTUB2,DDX24,ISG12(A),PPP4R4,SERPINA6
	7	115,550,783	rs80918930	4.21E-07 **	ISG12(A),PPP4R4,SERPINA6,SERPINA1,SERPINA11,UABP-2,SERPINA12
	7	115,571,143	rs80966458	4.21E-07 **	PPP4R4,SERPINA6,SERPINA1,SERPINA11,UABP-2,SERPINA12
	7	115,603,615	rs80873210	1.82E-07 **	PPP4R4,SERPINA6,SERPINA1,SERPINA11,UABP-2,SERPINA12,SERPINA4,SERPINA5
	7	115,616,606	rs80825941	1.82E-07 **	PPP4R4,SERPINA6,SERPINA1,SERPINA11,UABP-2,SERPINA12,SERPINA4,SERPINA5
	7	115,679,840	rs80953170	3.96E-07 **	PPP4R4,SERPINA6,SERPINA1,SERPINA11,UABP-2,SERPINA12,SERPINA4,SERPINA5
	7	115,749,343	rs80822022	1.31E-07 **	SERPINA6,SERPINA1,SERPINA11,UABP-2,SERPINA12,SERPINA4,SERPINA5
	7	115,760,859	rs80893020	1.31E-07 **	SERPINA1,SERPINA11,UABP-2,SERPINA12,SERPINA4,SERPINA5
	7	115,823,340	rs80810258	2.26E-07 **	SERPINA11,UABP-2,SERPINA12,SERPINA4,SERPINA5,SERPINA3-2
	7	115,973,303	rs80937516	1.90E-07 **	SERPINA4,SERPINA5,SERPINA3-2,GSC
	7	116,000,136	rs80948347	7.67E-08 **	SERPINA5,SERPINA3-2,GSC
	7	118,819,341	rs80869079	2.02E-05 *	-
	7	118,832,173	rs80849807	2.02E-05 *	-
	7	118,858,539	rs80792506	2.02E-05 *	-
	7	119,030,288	rs80804399	1.98E-05 *	-
	7	119,102,986	rs80852461	2.62E-05 *	-
	7	119,132,019	rs80936553	1.25E-05 *	-
	7	119,190,574	rs80867008	1.03E-05 *	-
	7	119,204,491	rs80848734	1.03E-05 *	-
	7	119,357,414	rs80865857	1.22E-05 *	-
	7	119,457,021	rs80978493	1.22E-05 *	-
	7	119,603,213	rs80876684	1.65E-05 *	-
	7	120,070,008	rs80846272	1.32E-05 *	BCL11B
	7	120,096,033	rs80871510	1.32E-05 *	BCL11B
	7	120,128,804	rs80994655	1.18E-05 *	BCL11B
	7	120,141,153	rs80796024	1.59E-05 *	BCL11B
IFN-γ	12	13,502,838	rs81440208	2.49E-06 *	CACNG5,CACNG4,CACNG1,HELZ
	12	13,710,118	rs81214705	9.02E-06 *	HELZ,PSMD12,PITPNC1
	12	13,799,478	rs81261131	9.02E-06 *	HELZ,PSMD12,PITPNC1
	12	13,826,063	rs81440273	9.02E-06 *	HELZ,PSMD12,PITPNC1
	12	13,846,528	rs81440280	8.57E-06 *	HELZ,PSMD12,PITPNC1,NOL11
	12	13,859,215	rs81440287	5.88E-06 *	PSMD12,PITPNC1,NOL11
	12	14,110,163	rs81440328	2.25E-05 *	PITPNC1,NOL11,BPTF,C17orf58,KPNA2
	12	14,204,868	rs81255563	1.23E-05 *	PITPNC1,NOL11,BPTF,C17orf58,KPNA2,SMURF2
	12	14,511,766	rs81216365	2.53E-05 *	SMURF2,CEP95,DDX5,POLG2,MILR1,PECAM1
	12	14,655,763	rs81440391	2.13E-05 *	SMURF2,CEP95,DDX5,POLG2,MILR1,PECAM1,TEX2
	12	14,709,054	rs80892486	1.85E-05 *	CEP95,DDX5,POLG2,MILR1,PECAM1,TEX2,ERN1
	12	14,732,256	rs81440339	2.63E-05 *	CEP95,DDX5,POLG2,MILR1,PECAM1,TEX2,ERN1
	12	14,747,002	rs81440416	2.65E-05 *	CEP95,DDX5,POLG2,MILR1,PECAM1,TEX2,ERN1

[a]Abbreviations of traits are shown in Table 2.

[b]SSC: Sus Scrofa chromosome.

[c]**: Genome-wide significant level. *: Genome-wide suggestive level.
