# Supplementary material for: Genome-wide association studies for production, respiratory disease, and immune-related traits in Landrace pigs
Source: Sci Rep. 2021 Aug 4;11:15823. doi: 10.1038/s41598-021-95339-2 (PMC8338966; doi:10.1038/s41598-021-95339-2)
Supplement: Supplementary file 6 — Supplementary Table S3. [file 41598_2021_95339_MOESM6_ESM.pdf]

Genome-wide association studies for production, respiratory disease, and immune-related traits in Landrace pigs

Yoshinobu Uemoto[1†*], Kasumi Ichinoseki[1†], Toshimi Matsumoto[2], Nozomi Oka[3], Hironori Takamori[3], Hiroshi Kadowaki[3], Chihiro Kojima-Shibata[3], Eisaku Suzuki[3], Toshihiro Okamura[4], Hisashi Aso[1], Haruki Kitazawa[1], Masahiro Satoh[1], Hirohide Uenishi[2], Keiichi Suzuki[1]

[1]Graduate School of Agricultural Science, Tohoku University, Sendai, Miyagi 980-8572, Japan.
[2]Animal Bioregulation Unit, Division of Animal Sciences, Institute of Agrobiological Sciences, National Agriculture and Food Research Organization (NARO), Tsukuba, Ibaraki 305-8634, Japan
[3]Miyagi Prefecture Animal Industry Experiment Station, Osaki, Miyagi 989-6445, Japan
[4]Institute of Livestock and Grassland Science, NARO, Tsukuba, Ibaraki 305-0901, Japan

[†]These authors have contributed equally to this work and share first authorship.
[*]Correspondence author

Supplementary table
Table S3. Significant gene ontology (GO) terms and Kyoto Encyclopedia of Genes and Genomes (KEGG) pathways associated with production and immune-related traits (*p*-value < 0.05).

Table S3. Significant gene ontology (GO) terms and Kyoto Encyclopedia of Genes and Genomes (KEGG) pathways associated with production and immune-related traits (*p* -value < 0.05)

Traits[a]	Database	Term ID	Term description	Counts	Invovled genes	DAVID *p* -value
Production traits						
TDG	GO	GO:0045893	Positive regulation of transcription, DNA-templated	5	MAP2K3, RAI1, USP22, TNNI2, SOX7	5.71E-03
	GO	GO:0015629	Actin cytoskeleton	3	MSRA, MPRIP, LSP1	3.57E-02
	GO	GO:0003779	Actin binding	4	MPRIP, TNNT3, TNNI2, LSP1	7.77E-03
BF	GO	GO:0010951	Negative regulation of endopeptidase activity	3	SERPINB12, SERPINB13, SERPINB5	8.99E-03
	GO	GO:0004867	Serine-type endopeptidase inhibitor activity	3	SERPINB12, SERPINB13, SERPINB5	5.24E-03
	GO	GO:0002020	Protease binding	3	SERPINB13, MBP, MALT1	5.67E-03
Immune-related traits						
WBC_105	GO	GO:0042826	Histone deacetylase binding	3	MEF2B, AKAP8L, AKAP8	1.59E-02
	GO	GO:0044822	Poly(A) RNA binding	7	BST2, UPF1, DDX49, AKAP8L, CCDC124, AKAP8, SUGP2	1.79E-02
RGL_105	GO	GO:0005829	Cytosol	6	NCF2, GMNN, ACOT13, ARPC5, RGL1, SMG7	6.82E-03
CORT_105	GO	GO:0010951	Negative regulation of endopeptidase activity	6	SERPINA11, SERPINA12, SERPINA1, SERPINA6, SERPINA4, SERPINA5	3.40E-08
	GO	GO:0005615	Extracellular space	6	SERPINA11, SERPINA12, SERPINA1, SERPINA6, SERPINA4, SERPINA5	2.49E-03
	GO	GO:0004867	Serine-type endopeptidase inhibitor activity	6	SERPINA11, SERPINA12, SERPINA1, SERPINA6, SERPINA4, SERPINA5	7.00E-09
IFN-γ	GO	GO:0061337	Cardiac conduction	3	CACNG1, CACNG4, CACNG5	4.20E-05
	GO	GO:0070588	Calcium ion transmembrane transport	3	CACNG1, CACNG4, CACNG5	2.96E-04
	GO	GO:0006810	Transport	3	CACNG1, CACNG4, CACNG5	2.50E-03
	GO	GO:0005245	Voltage-gated calcium channel activity	3	CACNG1, CACNG4, CACNG5	5.45E-05
	KEGG	hsa04010	MAPK signaling pathway	4	CACNG1, CACNG4, CACNG5, MAP2K6	1.91E-04
	KEGG	hsa05412	Arrhythmogenic right ventricular cardiomyopathy (ARVC)	3	CACNG1, CACNG4, CACNG5	5.54E-04
	KEGG	hsa04260	Cardiac muscle contraction	3	CACNG1, CACNG4, CACNG5	6.94E-04
	KEGG	hsa05410	Hypertrophic cardiomyopathy (HCM)	3	CACNG1, CACNG4, CACNG5	7.50E-04
	KEGG	hsa05414	Dilated cardiomyopathy	3	CACNG1, CACNG4, CACNG5	8.70E-04
	KEGG	hsa04261	Adrenergic signaling in cardiomyocytes	3	CACNG1, CACNG4, CACNG5	2.33E-03
	KEGG	hsa04921	Oxytocin signaling pathway	3	CACNG1, CACNG4, CACNG5	2.75E-03
TNF-α	GO	GO:0044822	Poly(A) RNA binding	3	LARP1, GEMIN5, MRPL22	2.45E-02
Multi-trait meta-analysis						
	GO	GO:0010951	Negative regulation of endopeptidase activity	5	SERPINA11, BST2, SERPINA12, SERPINA1, SERPINA6	5.30E-05
	GO	GO:0006914	Autophagy	3	MAP1S, MVB12A, CTSD	2.20E-02
	GO	GO:0004867	Serine-type endopeptidase inhibitor activity	4	SERPINA11, SERPINA12, SERPINA1, SERPINA6	4.87E-04

[a]Abbreviations of traits are shown in Table 2.
